# The Influence of Ultrafiltration of *Citrus limon* L. Burm. cv Femminello Comune Juice on Its Chemical Composition and Antioxidant and Hypoglycemic Properties

**DOI:** 10.3390/antiox8010023

**Published:** 2019-01-16

**Authors:** Monica Rosa Loizzo, Vincenzo Sicari, Rosa Tundis, Mariarosaria Leporini, Tiziana Falco, Vincenza Calabrò

**Affiliations:** 1 Department of Pharmacy, Health Science and Nutrition, University of Calabria, Via Pietro Bucci, 87036 Arcavacata di Rende (CS), Italy; monica_rosa.loizzo@unical.it (M.R.L.); mariarosarialeporini@tiscali.it (M.L.); tiziana.falco@unical.it (T.F.); 2 Department of Agricultural Science, Mediterranean University of Reggio Calabria, Via Graziella, Feo di Vito, 89123 Reggio Calabria, Italy; vincenzo.sicari@unirc.it; 3 Department of Computer Engineering, Modelling, Electronics and System Science (DIMES), University of Calabria, Via Pietro Bucci, 87036 Arcavacata di Rende (CS), Italy; vincenza.calabro@unical.it

**Keywords:** juice, membrane, flavonoids, biological properties, carbohydrates hydrolyzing enzymes

## Abstract

Membrane separation has brought about a significant change in the food processing industry because it could operate separation at low temperature without a reduction of nutrients and bioactive compounds. *Citrus limon* L. Burm. cv Femminello comune juice, an Italian IGP (Protected Geographical Indication) product, was subjected to the ultrafiltation (UF) process using a cellulose acetate membrane, with a cut-off of 100 kDa, subjected to different transmembrane pressures (TMP, 05–1.5 bar). Untreated and ultra-filtrated (UF) juices were investigated for physicochemical parameters including pH, titratable acidity (TA), total soluble solids (TSS) and ascorbic acid content. Total phenols (TPC) and flavonoids (TFC) contents were also determined. Rutin, hesperidin, eriocitrin, and neohesperidin were selected as markers and quantified by HPLC. Antioxidant potential was investigated by using DPPH, ABTS, and FRAP tests. RACI was used to identify the sample with highest antioxidant potential. The hypoglycemic activity was examined using carbohydrates hydrolyzing enzymes assay. The application of increasing pressures across the membrane led to a reduction in TSS without causing a loss of bioactive compounds in terms of TPC and TFC. UF juice obtained with TMP of 1.5 bar (J3) showed a significant amount of eriocitrin and hesperidin with concentrations of 15.8 and 10.5 mg/100 mL, respectively. This sample showed the highest antioxidant potential and exhibited a promising α-amylase and α-glucosidase inhibitory activity with IC_50_ values of 31.1 and 35.3 mg/mL, respectively. Collectively our results support the use of cellulose acetate membrane to obtain an ultra-filtered juice with significant health potential.

## 1. Introduction

The development of separative membranes has been particularly important in the food processing industry [1,2]. The membranes exploit the different chemical-physical properties of the mixtures to obtain the separation of the components at low temperature and with less energy use.

Among the membrane-based separating techniques, ultrafiltration (UF) is one of the most widely used methods, especially for fruit juice clarification. UF juice has a longer shelf-life due to a lack of pectin [3,4,5,6].

*Citrus* species are a rich source of bioactive products including flavonoids, phenols, ascorbic acid and terpenes, and are well-known for their health-promotion values [7,8,9]. *Citrus limon* fruits are the main fruit trees grown throughout the world. *Citrus* is primarily valued for the fruits, which is either eaten alone as fresh fruit, processed into juice, or added to dishes and beverages.

*Citrus* fruits possess several biological properties including antioxidant, anti-inflammatory antiviral, and anticancer activities, effects on platelet aggregation and capillary fragility. More recently, therapeutic values related to cardiovascular diseases and age related macular degeneration have been reported. These health properties are linked to the high amounts of phytochemicals and in particular flavonoids available in this fruit [10]. Due to the popularity of health effects and in particular the antioxidant activity upon intake of fresh fruits, the demand for fresh fruit juices has rapidly increased. In this context, *Citrus* juices have received much attention because of their nutritional and antioxidant properties and because nowadays the prevention of health problems through nutrition is promoted intensively [11].

Oxidative stress is a complex reaction in which Radical Oxigen Species (ROS) are involved. Wright et al. [12] showed that an ROS excess can cause β-cell dysfunction, impaired glucose tolerance and type 2 diabetes mellitus (T2DM). Moreover, ROS have also been implicated in the progression of T2DM complications, including vascular dysfunction. Additionally, the excess of glucose in plasma determined a reaction between glucose and proteins with formation of glycation end-products, triggering production of ROS [13]. With the postulation that hyperglycaemia leads to generation of ROS, it follows that a treatment able to counteract oxidative stress and post-prandial glucose peak is a suitable strategy to prevent this disease.

The present study was designed to test the effect of ultrafiltration membrane processing on chemical composition and antioxidant activity of *C. limon* from Rocca Imperiale (Calabria, Italy).

For this purpose, total phenols and flavonoids contents were measured. Rutin, hesperidin and neohesperidin were selected as markers and quantitatively analyzed by HPLC. The radical scavenging activity was investigated by DPPH and ABTS tests. FRAP test was also performed. The hypoglycemic effects were determined by using α-amylase and α-glucosidase inhibition tests.

## 2. Materials and Methods

### 2.1. Chemicals and Reagents

Reagents used in this study were obtained from Sigma-Aldrich S.p.a. (Milan, Italy). Solvents of analytical grade were purchased from VWR International s.r.l. (Milan, Italy). Acarbose from *Actinoplanes* sp. was obtained from Serva (Heidelberg, Germany). Rutin, hesperidin, eriocitrin, and neohesperidin were purchased from Extrasynthese (Genay-France).

### 2.2. Plant Material and Juice Preparation

Fruits from *Citrus limon* L. Burm. were collected in Rocca Imperiale (Latitude: 40.1108, Longitude: 16.5811, Calabria, South of Italy) during December 2012. These fruits belong to a spontaneous mutation of the cultivar “Femminello commune”. A set of fruits representative of this cultivar was obtained harvesting fruits at full fruit size at maturity from 20 plants. Fruits were examined for integrity and absence of dust and insect contamination, were peeled and devoid of seeds and squeezed with domestic squeezer to obtain the juice that is characterized by a yellow citrine color. Juice was stored at 4 °C prior to analysis.

### 2.3. Ultrafiltration Process

*C. limon* juice was processed in a pilot ultrafiltration (UF) system (Osmonics, MN, USA). The system consisted of a 200 mL stainless steel feed tank, a feed pressure pump, a pressure control system, a thermometer, a feed flow meter, two manometers for the measure of the inlet and outlet pressures. A data acquirement system connected to the system allows monitoring of the transmembrane pressure and the axial feed flow rate. The permeate fluxes were measured by using a digital balance connected to the system. Moreover, the UF system was equipped with a flat sheet regenerated cellulose membrane (C100F) supplied by the NADIR Company (MICRODYN-NADIR, Wiesbaden, Germany) with a cut-off of 100 kDa and membrane surface area of 44.0 cm^2^. Experiments were performed according to the total recycle and the batch concentration mode. In the batch concentration mode, UF system was operated at three different transmembrane pressures of 0.5, 1.0 e 1.5 bar and at a temperature of 25 °C. For membrane preparation, the module was rinsed with distilled water for 10 min three times before the use of the juice.

### 2.4. Physiochemical Characteristics of C. Limon Juice

*C. limon* juice was weighed and the percentage of juice was estimated. The total soluble solids (TSS) was determined using a Digital Hand-Held Pocket Refractometer (Atago, Milan, Italy) with scale range of 0–32° Brix [14]. pH was measured by using an Orion Star™ A211 pH Benchtop Meter (Fischer Scientific, Milan, Italy). The titratable acidity (TA) determination was done using a NaOH standard solution and phenolphthalein indicator. Results are expressed as a percentage of citric acid using the formula of American Organization Analytical Chemists (AOAC) [15]: acid % = (0.1 × 0.064 × V in mL of NaOH used × 100)/mL of the sample juice.

### 2.5. Ascorbic Acid Content

The content of ascorbic acid was evaluated using the method described in AOAC [15], with some slight modifications. 50 mL of juice and 50 mL of oxalic acid (4%) were mixed. 10 mL of this mixture was treated with 2,6-dichlorophenol indophenol dye as indicator. The ascorbic acid content is expressed in mg/100mL of juice according to the following formula: Ascorbic acid (mg/100 mL) = [Titer × Dye factor × Volume made up × 100]/[Aliquot of sample used × Volume of sample].

### 2.6. Total Phenols and Flavonoids Content

The Folin–Ciocalteu method was used to determine the total phenols content as previously described [16]. Briefly, sample was mixed with Folin–Ciocalteu reagent (0.2 mL), 15% Na_2_CO_3_ (1 mL), and distilled water (2 mL). The absorbance was measured at 765 nm after 2 h of incubation by using a UV–Vis Jenway 6003 spectrophotometer (Carlo Erba, Milan, Italy). The total phenols content is reported as mg gallic acid equivalents/100 mL of juice (Table 1).

The total flavonoids content was evaluated as previously described [17]. The absorbance was measured at 510 nm. The total flavonoids content is expressed as mg quercetin equivalents/100 mL of juice (Table 1).

### 2.7. HPLC-DAD Analyses

Rutin, hesperidin, eriocitrin and neohesperidin selected as marker were quantified by HPLC. HPLC analyses were realized using an HPLC system HP 1100 equipped with two pumps, DAD (Diode Array Detector) detector (280 nm) (Agilent, Cernasco sul Naviglio, Milan, Italy), column oven, injector and a C18 RP column (Phenomenex Luna 5 lm C18, 250 × 4.60 mm) (Phenomenex, Inc., Bologna, Italy). The column temperature was 30 °C. The mobile phase was H_2_O/formic acid (0.1%) (A) and acetonitrile (B) with a flow rate of 1 mL/min (2 min, 100%). The elution gradient started with 100% A for 2 min, increased to 100% B for 2 min to 30 min followed by a return to 100% A in 5 min, and a final isocratic part with 100% A to 36 min.

### 2.8. Antioxidant Effects

#### 2.8.1. DPPH (2,2-diphenyl-1-picrylhydrazyl) Assay

The DPPH radical scavenging assay was applied using the procedure previously described [18]. The DPPH solution (1.0 × 10^−4^ M) and treated and UF juice at different concentrations (62.5–100 µg/mL) were mixed. The absorbance was measured at 517 nm against blank without DPPH reagent. The DPPH radical-scavenging activity was calculated following the equation: DPPH radical-scavenging activity (%) = [1−(sample absorbance with DPPH–sample absorbance without DPPH)] × 100. Results are reported as IC_50_ values (μg/mL).

#### 2.8.2. ABTS, 2,2′-azino-bis(3-ethylbenzothiazoline-6-sulphonic acid) Assay

The ABTS method was employed following the procedure previously reported [18]. A 7 mM ABTS solution with 2.45 mM potassium persulphate was used to produce ABTS radical. Afterwards, the solution was diluted with ethanol until an absorbance of 0.70 ± 0.05 read at 734 nm was obtained. Successively, 25 μL of sample at concentrations ranging from 25 to 400 μg/mLwas added to 2 mL of ABTS ethanolic solution and left to react for 6 min at room temperature. The ABTS radical scavenging activity was calculated following the equation: ABTS scavenging activity (%) = [(A_0_ – A)/A_0_] × 100 where A_0_ is the absorbance of the control reaction and A is the absorbance in the presence of samples. Ascorbic acid was used as positive control. Results are reported as IC_50_ values (μg/mL).

#### 2.8.3. Ferric Reducing Ability Power (FRAP) Assay

The FRAP test measures the ability of antioxidants to induce the reduction of TPTZ (2,4,6-tripyridyl-s-triazine)-Fe^3+^. This assay was performed using the procedure previously described [19]. In brief, a FRAP mixture of 2.5 mL of 10 mM tripyridyltriazine (TPTZ) solution in 40 mM HCl, 2.5 mL of 20 mM FeCl_3_ and 25 mL of 0.3 M acetate buffer (pH 3.6) was prepared. Untreated and UF juice (2.5 mg/mL) was dissolved with 1.8 mL of FRAP reagent. The absorbance was read at 595 nm. FRAP value is expressed as μM Fe(II)/g. Butylated hydroxytoluene (BHT) was used as a positive control.

### 2.9. RACI Calculation

Relative Antioxidant Capacity Index (RACI) is a statistical application useful to estimate the antioxidant ability of samples from different in vitro tests [19]. The standard score is calculated by using the following equation: (x−μ)/σ, where x is the raw data, μ is the mean, and σ is the standard deviation.

### 2.10. In Vitro Hypoglycaemic Activity

The α-amylase method was performed following the procedure previously described [18]. A solution with starch, α-amylase enzyme and 3,5 dinitrosalicilic acid was prepared. Both untreated and UF juices were added to this solution and left to incubate for 5 min at 25 °C. The absorbance was read at 540 nm. Acarbose was used as positive control. For the inhibition of α-glucosidase, the methodology previously described by Loizzo et al. [18] was used. In the first step of experiment, a solution of maltose and *o*-dianisidine was prepared. Both untreated and UF juices were added to this solution and left to incubate for 5 min at 37 °C. In the second step, the enzyme was added to the solution and left to incubate for 30 min at the same temperature. The stop of the enzymatic reaction was obtained by using perchloric acid. The supernatant of tube of step one was mixed with the colorant *o*-dianisidine and peroxidase/glucose oxidase (PGO) and left to incubate for 30 min at 37 °C. After this time, absorbance was read at 500 nm. Acarbose was used as positive control.

### 2.11. Statistical Analysis

Experiments were performed in triplicate. Data are expressed as means ± standard deviation (S.D.). Prism GraphPad Prism version 4.0 for Windows (GraphPad Software, San Diego, CA, USA) was used to calculate sample concentration that determined a 50% of inhibition (IC_50_). An analysis of variance (ANOVA) was performed using SPSS 17.0 (SPSS inc., Chicago, IL, USA) to determine significant differences among the treatments. Significant differences were calculated using Tukey’s post-hoc tests. Differences at *p* < 0.05 were considered to be statistically significant and at *p* < 0.01 were considered to be highly significant. A multicomparison Dunnett’s test was applied in order to investigate the differences between sample and positive control in all bioassays (*p < 0.01).

## 3. Results and Discussion

### 3.1. Juice Content and Physicochemical Parameters

*C. limon* from Rocca Imperiale was squeezed to obtain a percentage of juice of 45.2% (Table 1).

**Figure 1:**
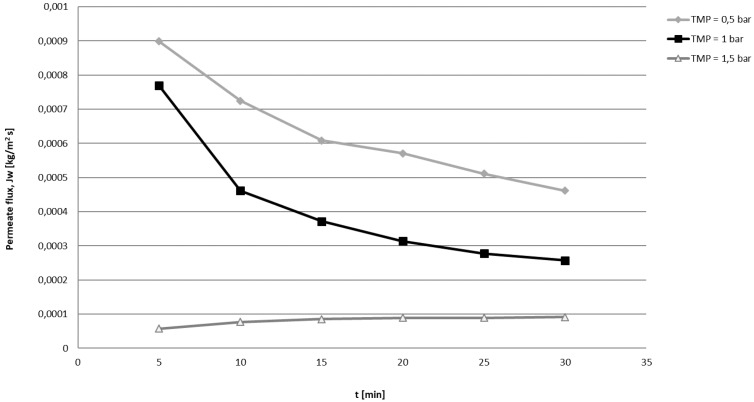
Permeate flux as function of time at different TMP.

This juice content is in line with those reported by the IGP document (juice content > 30%) and in line with *C. limon* juice content that is in the range 30.22% for Monachello to 58.42 for Meyer variety [20]. Our juice showed a content similar to Santa Tereza variety (46.27%) [20]. Pearmeate flux as function of time monitored at three different TMP is reported in Figure 1.

The difference between the treated and untreated samples was statistically significant (*p* < 0.05) as reported in Table 1. pH, citric acid and ascorbic acid contents were not significantly affected by TMP as reported in Table 1. A lower pH was recorded in juice from *C. limon* Santa Tereza variety (Ph = 2.21) [20]. As expected, UF determined a reduction of TSS mainly at TMP of 1.5 bar (2.7 °Brix) vs. untreated juice (8.5 °Brix).

### 3.2. Bioactive Compounds

*C. limon* juice is a promising source of bioactive constituents with 151.7 and 20.8 mg/100 mL of TPC and TFC, respectively (Table 2). The ultrafiltration process indicated that as the applied pressure increased, an enrichment of permeate in phenols and total flavonoids was observed. Previously, Espamer et al. [21] analyzed the effect of microfiltration on *C. lemon* juice quality and proved that permeate juice presented a TA, pH and TSS comparable to the untreated juice and with an optimal TMP of 0.6 bar. More recently, Chornomaz et al. [22] showed that clarification of lemon juice with membrane prepared with tubular polyvinylidene fluoride and polyvinylpyrrolidone produced a product similar to the natural one and with a good permeate flux.

A clarified orange juice with similar parameters to our untreated juice, except for TSS, was obtained by Cassano et al. [23]. In this case a tubular polyvinylidene difluoride (PVDF) membrane with a cut-off of 15 KDa was used. The UF juice was characterized also by a promising polyphenols content and total antioxidant activity. In the clarification of *Citrus* juices excellent performances have also been obtained with PVDF/PMMA with a TMP of 1 bar obtaining the complete removal of soluble solid [24].

HPLC analysis quantified rutin, hesperidin, eriocitrin, and neohesperidin. Eriocitrin and hesperidin are the two main abundant compounds of untreated juice with concentrations of 16.7 and 14.1 mg/100 mL juice, respectively. Differences between treated and untreated samples were statistically significant (*p* < 0.01). Among permeate juices, the sample treated with a TMP of 1.5 bar contained the higher amount of these compounds with concentrations of 15.8 and 10.5 mg/100 mL juice for eriocitrin and hesperidin, respectively. Rutin and neohesperidin are contained in concentrations of 2.9 and 6.0 mg/100 mL juice, and 2.8 and 5.7 for untreated juice and J3, respectively. Generally, the application of increasing pressures caused an increase in the presence of the markers in the permeate due to a forced passage through the membrane. Our data are in agreement with those reported by Gattuso et al. [25] in which eriocitrin and hesperidin obtained a mean of 16.7 and 20.5 mg/100 mL juice, respectively. These values are higher than that found in commercial lemon juice where a mean of 16.0, 7.07 and 1.45 mg/100 mL juice were found for eriocitrin, hesperidin and neohesperidin, respectively. The differences between natural and commercial lemon juice flavonoids composition was investigated also by Hajimahmoodi et al. [26]. Thirty-eight natural and sixty-two branded lemon juices were injected in HPLC. Eriocitrin and hesperindin were found in the range 3.24–10.68 and 49.06–104.84 μg/mL, respectively. The comparison of data from literature about the flavonoids composition of *Citrus* species indicated that *C. limon* was characterized by a moderate flavonoids content independently of the varieties and was dominated by hesperidin and eriocitrin [27]. A lower TPC content of 0.322 mg GAE per 100 mL of juice was found by Fejzić & Ćavar [28].

### 3.3. Antioxidant Activity

Herein, the antioxidant activity of untretated juice and ultrafiltrated juices (J1–J3) was investigated and compared. A concentration-effect relationship was found for all extracts in all tests, except for FRAP test (Table 3). The radical scavenging activity was assessed by using ABTS and DPPH assay. Due to the different nature of radicals, a different response to antioxidant compounds could be registered. Moreover, researchers recommend the use of different methodologies in order to obtain more reliable data [29].

As reported in Table 3, juice UF at TMP of 1.5 bar showed the best antioxidant activity with IC_50_ values of 35.2 and 37.3 μg/mL for DPPH and ABTS, respectively. Sample J1 showed the highest FRAP value (52.7 μM Fe(II)/g).

The statistical approaches RACI was used in order to recognize samples with the highest antioxidant activity. As evidenced in Table 3, sample J3 showed the highest antioxidant potency.

A great variability in FRAP values (29.58–660.13 μg/mL) were found when natural and commercial lemon juice were tested [26]. A higher FRAP value of 122.75 μM Fe(II)/g was recorded for concentrated lemon juice [30]. Recently, Xi et al. [31] compared the phenolic composition and antioxidant potential of seed, peel, whole fruits and juice of different *C. limon* (Feiminailao, Cuningmeng Limeng, Pangdelusaningmeng, Beijingningmeng) and showed that peels and whole fruit had significantly higher levels of phenols than other investigated portions. In particular, Cuningmeng Limeng, Pangdelusaningmeng varieties contained the higher phenols content together with the higher antioxidant potential.

### 3.4. Carbohydrate Hydrolysing Enzymes Inhibitory Activities

In this work, the inhibitory activity of juice and UF juice on α-amylase and α-glucosidase was investigated. Inhibitors of both enzymes are commonly used for the treatment of hyperglycemic conditions due to the blocking of carbohydrates breakdown in the small intestine [32]. All samples are able to inhibit both enzymes in a concentration dependent manner (Table 4).

UF juice obtained with a transmembrane pressure of 1.5 bar showed the highest inhibitory activity with IC_50_ values of 31.1 and 35.5 μg/mL for α-amylase and α-glucosidase, respectively. Both values are lower than that found for acarbose (positive control). 

Correlation analysis revealed that neither of identified markers are alone responsible of the hypoglycemic effect.

Several research articles showed that members of *Citrus* family are able to regulate glycaemia. Percentages of 75.55 and 70.68% were found against α-amylase and α-glucosidase by *C. hystrix* juice, whereas *C. maxima* showed percentages of inhibition of 79.75 and 72.83% against α-amylase and α-glucosidase, respectively [33].

Data obtained with *C. limon* juice are better than that obtained in our previous investigation with *C. clementina* juice in which IC_50_ values of 226.6-243.2 and 77.8–200.9 μg/mL for α-amylase and α-glucosidase, respectively, were found [15]. Riaz et al. [34] evidenced that *C. limon* juice administration (0.2–0.6 mL/kg per day) decreased blood glucose level in a dose-dependent manner. A potent hypoglycemic effect was also demonstrated for bergamot juice administered for 30 days in 237 patients [35]. Moreover, Hamed et al. [36] revealed that administration of 0.5 mL of *C. paradisi* juice twice a day for 12 days determined a significant decrease of serum glucose level in both normo-glycemic and diabetic rats. The hypoglycemic activity of *Citrus* juice was confirmed by Mallick & Khan [37] that compared the effect of *C. sinensis* and *C. paradisi* at three different doses (0.1, 0.3 and 0.5 mL/kg). *C. sinensis* determined a significant reduction of blood glucose level and rise in plasma insulin level at lowest concentration of 0.1 mL/kg. A similar effect required administration of *C. paradisi* at 0.5 mL/kg. A significant effect on glycemic control was observed when combination of *C. sinensis* and *C. paradisi* juices were administered in alloxan-induced diabetic rats.

Among phytochemicals identified in *Citrus*, flavonoids are considered responsible for the hypoglycemic effect of these species. These phytochemicals are able not only to inhibit α-amylase and α-glucosidase enzymes but also to inhibit sodium-dependent glucose transporter 1 (SGLT1), enhance insulin-dependent glucose uptake and at the same time reduce hepatic glucose output, and stimulate insulin secretion [33,38,39].

Untreated and UF juices contained rutin, eriocitrin, hesperidin and neohesperidin. Hesperidin exhibited both α-glucosidase and α-amylase inhibitory activity with IC_50_ values of 15.89 and 26.04 μM and together with neohesperidin are able to inhibit amylase-catalyzed starch digestion [33].

Moreover, Sahnoun et al. [40] demonstrated that hesperidin has 51 polar contact with the enzyme-binding site and that this contact is higher than that found for acarbose. Neohesperidin was also able to reduce serum glucose and glycosylated serum protein in vivo [41]. Also, rutin demonstrated to inhibit both α-glucosidase and α-amylase with IC_50_ values of 0.037 and 0.043 μM, respectively [42].

## 4. Conclusions

The application of cellulose acetate membrane for ultrafiltration of *C. limon* juice determined a significant reduction of TSS as requested by the relevant industries and consumers without affecting the healthy properties of the juice. In fact, at maximum TMP applied to the system, the resulting permeate showed a promising antioxidant activity due to the preserved ascorbic acid, and the TPC and TFC content. It also showed significant carbohydrate hydrolyzing enzyme inhibitory activities due to the high content of flavonoids hesperidin and neohesperidin.

## Figures and Tables

**Table 1:** Effect of different pressure on juice physicochemical parameters.

Juice	P (bar)	Juice (%)	pH % Inhibition	TSS (°Brix) µM Fe(II)	% Citric Acid	Ascorbic Acid Content (mg/100 mL)
Untreated	0	45.2 ± 2.6	2.5 ± 0.01 ^c^	8.5 ± 2.1 ^a^	6.3 ± 1.8 ^a^	30.8 ± 2.7 ^a^
J1	0.5	-	2.6 ± 0.02 ^b^	7.8 ± 2.4 ^b^	5.6 ± 2.0 ^c^	27.7 ± 3.1 ^d^
J2	1	-	2.7 ± 0.03 ^a^	3.5 ± 2.2 ^c^	5.7 ± 2.1 ^b^	28.6 ± 2.5 ^c^
J3	1.5	-	2.6 ± 0.01 ^b^	2.7 ± 2.4 ^d^	5.7 ± 2.2 ^b^	29.4 ± 3.0 ^b^

Values are expressed as means ± standard deviation of triplicates. The lowercase letters represent the significant difference vertically at *p* < 0.05.

**Table 2:** Effect of different pressure on TPC, TFC and selected markers quantified by HPLC.

Juice	TPC ^a^	TFC ^b^	Rutin ^c^	Eriocitrin ^c^	Hesperidin ^c^	Neohesperidin ^c^
Untreated	151.7 ± 3.4 ^a^	30.8 ± 2.5 ^a^	2.9 ± 0.9 ^a^	16.7 ± 1.9 ^a^	14.1 ± 1.8 ^a^	6.0 ± 0.3 ^a^
J1	102.0 ± 1.3 ^d^	19.7 ± 3.5 ^c^	2.2 ± 0.9 ^b^	14.8 ± 1.4 ^b^	12.6 ± 1.3 ^b^	5.0 ± 0.7 ^b^
J2	126.4 ± 4.9 ^c^	22.4 ± 3.0 ^b^	2.3 ± 0.8 ^b^	15.2 ± 1.3 ^b^	12.1 ± 1.5 ^b^	5.6 ± 1.2 ^b^
J3	148.4 ± 3.1 ^b^	31.9 ± 4.1 ^a^	2.8 ± 0.7 ^a^	15.8 ± 1.5 ^ba^	13.5 ± 1.3 ^ba^	5.7 ± 1.0 ^ba^

TPC: total phenols content; TFC: total flavonoids content; ^a^: mg Gallic Acid Equivalents/100 mL of juice; ^b^: mg quercetin equivalents /100 mL of juice; ^c^: mg/100 mL juice. Values are expressed as means ± standard deviation of triplicates. The lowercase letters represent the significant difference vertically at *p* < 0.05.

**Table 3:** Antioxidant activity of untreated and UF *C. limon* juice.

Juice	DPPH Test (IC_50_ g/mL)	ABTS Test (IC_50_ g/mL)	FRAP Test ^a^ (M Fe(II)/g)	RACI Values
	(IC_50_ μg/mL)	(IC_50_ μg/mL)	(μM Fe(II)/g)	
Untreated	40.3 ± 1.0 *	46.5 ± 1.2 *	49.7 ± 2.8 *	0.06
J1	42.1 ± 1.3 *	51.3 ± 1.6 *	52.7 ± 2.0 *	0.68
J2	41.2 ± 4.0 *	39.7 ± 1.1 *	49.8 ± 1.6 *	−0.09
J3	35.2 ± 1.0 *	37.3 ± 1.4 *	48.9 ± 1.5 *	−0.66
Ascorbic acid	5.0 ± 0.8	1.7 ± 0.9		
BHT			63.2 ± 4.3	

DPPH: 2,2-diphenyl-1-picrylhydrazyl; ABTS: 2,2′-azino-bis(3-ethylbenzothiazoline-6-sulphonic acid); FRAP: Ferric Reducing Ability Power; RACI: Relative Antioxidant Capacity Index. Data are given as media ± S.D. (*n* = 3); ^a^ at 2.5 mg/mL. Ascorbic acid and BHT are used as positive controls. Differences within and between groups were evaluated by one-way analysis of variance test followed by a multicomparison Dunnett’s test: * *p* < 0.01 compared with the positive control.

**Table 4:** Hypoglycemic activity of untreated and UF *C. limon* juices.

Juice	α-Amylase ^a^ (IC_50_ µL/mL)	α-Glucosidase ^a^ % Inhibition
	(IC_50_ μg/mL)	(IC_50_ μg/mL)
Untreated	40.3 ± 1.0 *	46.5 ± 1.2 *
J1	42.1 ± 1.3 *	51.3 ± 1.6 *
J2	41.2 ± 4.0 *	49.7 ± 1.1 *
J3	31.1 ± 1.0 *	35.3 ± 1.4 *
Acarbose	50.0 ± 1.4	35.5 ± 1.1

Data are given as media ± S.D. (*n* = 3); Acarbose was used as positive control. Differences within and between groups were evaluated by one-way analysis of variance test followed by a multicomparison Dunnett’s test: * *p* < 0.01 compared with the positive control.

## References

[1] Mulder M. (1991). Basic Principles of Membrane Technology.

[2] Tundis R., Loizzo M.R., Bonesi M., Sicari V., Ursino C., Manfredi I., Conidi C., Figoli A., Cassano A. (2018). Concentration of bioactive compounds from elderberry (*Sambucus nigra* L.) juice by nanofiltration membranes. Plant Foods Hum. Nutr..

[3] Girard B., Fukumoto L.R. (1999). Apple juice clarification using microfiltration and ultrafiltration polymeric membranes. LWT.

[4] Wang B.J., Wei T.C., Yu Z.R. (2005). Effect of operating temperature on component distribution of West Indian cherry juice in a microfiltration system. LWT.

[5] Razi B., Aroujalian A., Raisi A., Fathizadeh M. (2011). Clarification of tomato juice by cross-flow microfiltration. Int. J. Food Sci. Technol..

[6] Cassano A., Drioli E., Galaverna G., Marchelli R., Disilvestro G., Cagnasso P. (2003). Clarification and concentration of citrus and carrot juices by integrated membrane processes. J. Food Eng..

[7] Tundis R., Loizzo M.R., Bonesi M., Menichini F., Mastellone V., Colica C., Menichini F. (2012). Comparative study on the antioxidant capacity and cholinesterase inhibitory activity of *Citrus aurantifolia* Swingle, *C. aurantium* L., and *C. bergamia* Risso and Poit. peel essential oils. J. Food Sci..

[8] Tundis R., Loizzo M.R., Menichini F. (2014). An overview on chemical aspects and potential health benefits of limonoids and their derivatives. Crit. Rev. Food Sci. Nutr..

[9] Loizzo M.R., Tundis R., Bonesi M., Menichini F., De Luca D., Colica C., Menichini F. (2012). Evaluation of *Citrus aurantifolia* peel and leaves extracts for their chemical composition, antioxidant and anti-cholinesterase activities. J. Sci. Food Agric..

[10] Okwu D.E. (2008). *Citrus* fruits: A rich source of phytochemicals and their roles in human health. Int. J. Chem. Sci..

[11] Del Caro A., Piga A., Vacca V., Agabbio M. (2004). Changes of flavonoids, vitamin C and antioxidant capacity in minimally processed citrus segments and juices during storage. Food Chem..

[12] Wright E., Scism-Bacon J.L., Glass L.C. (2006). Oxidative stress in type 2 diabetes: The role of fasting and postprandial glycaemia. Int. J. Clin. Pract..

[13] Selim F., Wael A., Keith E.J. (2017). Diabetes-induced reactive oxygen species: Mechanism of their generation and role in renal injury. J. Diabetes Res..

[14] Pareek S., Paliwal R., Mukherjee S. (2010). Effect of juice extraction methods and processing temperature-time on juice quality of Nagpur mandarin (*Citrus reticulata* Blanco) during storage. J. Food Sci. Technol..

[15] Association of Official Analytical Chemists (AOAC) (2000). Official Methods of Analysis.

[16] Gao X., Ohlander M., Jeppsson N., Björk L., Trajkovski V. (2000). Changes in antioxidant effects and their relationship to phytonutrients in fruits of Sea buckthorn (*Hippophae rhamnoides* L.) during maturation. J. Agr. Food Chem..

[17] Yoo K.M., Lee C.H., Lee H., Moon B.K., Lee C.Y. (2008). Relative antioxidant and cytoprotective activities of common herbs. Food Chem..

[18] Loizzo M.R., Leporini M., Sicari V., Falco T., Pellicanò T., Tundis R. (2017). Investigating the in vitro hypoglycaemic and antioxidant properties of *Citrus × clementina* Hort. juice. Eur. Food Res. Technol..

[19] Loizzo M.R., Tundis R., Bonesi M., Menichini F., Mastellone V., Avallone L., Menichini F. (2012). Radical scavenging, antioxidant and metal chelating activities of *Annona cherimola* Mill. (cherimoya) peel and pulp in relation to their total phenolic and total flavonoid contents. J. Food Comp. Anal..

[20] Al-Mouei R., Wafaa C. (2014). Physiochemical juice characteristics of various *Citrus* species in Syria. J. Plant Nutr. Soil Sci..

[21] Espamer L., Pagliero C., Ochoab A., Marchese J. (2006). Clarification of lemon juice using membrane process. Desalination.

[22] Chornomaz P.M., Pagliero C., Marchese J., Ochoa N.A. (2013). Impact of structural and textural membrane properties on lemon juice clarification. Food Bioprod. Process..

[23] Cassano A., Marchio M., Drioli E. (2007). Clarification of blood orange juice by ultrafiltration: Analyses of operating parameters, membrane fouling and juice quality. Desalination.

[24] Pagliero C., Ochoa N.A., Marchese J. (2011). Orange juice clarification by microfiltration: Effect of operational variables on membrane fouling. Latin Am. Appl. Res..

[25] Gattuso G., Barreca D., Gargiulli C., Leuzzi U., Caristi C. (2007). Flavonoid composition of *Citrus* juices. Molecules.

[26] Hajimahmoodi M., Moghaddam G., Mohsen M., Sadeghi S., Oveisi N., Reza M., Behrooz J. (2014). Total antioxidant activity, and hesperidin, diosmin, eriocitrin and quercetin contents of various lemon juices. Trop. J. Pharm. Res..

[27] Peterson J.J., Beecher G.R., Bhagwat S.A., Dwyer J.T., Gebhardt S.E., Haytowitz D.B., Holden J.M. (2006). Flavanones in grapefruit, lemons, and limes: A compilation and review of the data from the analytical literature. J. Food Compost. Anal..

[28] Fejzić A., Ćavar S. (2014). Phenolic compounds and antioxidant activity of some citruses. Bull. Chem. Technol. Bosn. Herzeg..

[29] Antolovich M., Prenzler P.D., Patsalides E.S., Mc Donald S., Robards K. (2002). Methods for testing antioxidant activity. Analyst.

[30] Oikeh E.I., Omoregie E.S., Oviasogie F.E., Oriakhi K. (2015). Phytochemical, antimicrobial, and antioxidant activities of different citrus juice concentrates. Food Sci. Nutr..

[31] Xi W., Lu J., Qun J., Jiao B. (2017). Characterization of phenolic profile and antioxidant capacity of different fruit part from lemon (*Citrus limon* Burm.) cultivars. J. Food Sci. Technol..

[32] Matsui T., Ogunwande I.A., Abesundara K.J.M., Matsumoto K. (2006). Anti-hyperglycemic potential of natural products. Med. Chem..

[33] Tundis R., Bonesi M., Sicari V., Pellicanò T.M., Tenuta M.C., Leporini M., Menichini F., Loizzo M.R. (2016). *Poncirus trifoliata* (L.) Raf.: Chemical composition, antioxidant properties and hypoglycaemic activity via the inhibition of α-amylase and α-glucosidase enzymes. J. Funct. Foods.

[34] Riaz A., Alam Khan R., Ahmed M. (2013). Glycemic response of *Citrus limon*, pomegranate and their combinations in alloxan-induced diabetic rats. Aust. J. Basic Appl. Sci..

[35] Mollace V., Sacco I., Janda E., Malara C., Ventrice D., Colica C., Visalli V., Muscoli S., Ragusa S., Muscoli C. (2011). Hypolipemic and hypoglycaemic activity of bergamot polyphenols: From animal models to human studies. Fitoterapia.

[36] Hamed W.M.A., Abid K.Y., Al-Amin S.A.U. (2008). Hypoglycemic and hypolipidemic effects of grapefruit juice in diabetic rats. Tikrit J. Pure Sci..

[37] Mallick N., Khan R. (2015). Effect of *Citrus paradisi* and *Citrus sinensis* on glycemic control in rats. Afr. J. Pharm. Pharmacol..

[38] Tadera K., Minami Y., Takamatsu K., Matsuoka T. (2010). Inhibition of alpha-glucosidase and alpha-amylase by flavonoids. J. Nutr. Sci. Vitaminol..

[39] Kim Y., Keogh J.B., Clifton P.M. (2016). Polyphenols and glycemic control. Nutrients.

[40] Sahnoun M., Trabelsi S., Bejar S. (2017). *Citrus* flavonoids collectively dominate the α-amylase and α-glucosidase inhibitions. Biologia.

[41] Jia S., Hu Y., Zhang W., Zhao X., Chen Y., Sun C., Li X., Chen K. (2015). Hypoglycemic and hypolipidemic effects of neohesperidin derived from *Citrus aurantium* L. in diabetic KKA(y) mice. Food Funct..

[42] Oboh G., Ademosun A.O., Ayeni P.O., Omojokun O.S., Bello F. (2015). Comparative effect of quercetin and rutin on α-amylase, α-glucosidase, and some pro-oxidant-induced lipid peroxidation in rat pancreas. Comp. Clin. Pathol..

